# Development and Use a Novel combined in-vivo and in-vitro Assay for Anti-inflammatory and Immunosuppressive Agents

**Published:** 2013

**Authors:** Tan Li, Hong Chen, Xin Mei, Na Wei, Bo Cao

**Affiliations:** a *Department of Immunology, Medical College of the Chinese People’s Armed Police Force, Tianjin, People’s Republic of China.*; b *Tianjin Key Laboratory for Biomarkers of Occupational and Environmental Hazard, Medical College of the Chinese People’s Armed Police Force, Tianjin, People’s Republic of China.*; c *Department of Pharmacognosy, Medical College of the Chinese People’s Armed Police Force, Tianjin, People’s Republic of China.*

**Keywords:** Immunoregulation, Delayed hypersensitivity, T lymphocytes, *in-vitro*

## Abstract

Contact hypersensitivity (CHS) mouse model induced by 2, 4-dinitrofluorobenzene (DNFB) is thought to be a T helper 1 (Th1)-dominant response and used for investigating anti-inflammatory and immunosuppressive agents. However, it is hardly used for screening large-scale drugs because of the large number of animals and complex mechanisms involved *in-vivo*. In this study, we focused on whether T lymphocytes from CHS mouse model could maintain the state of immune response *in-vitro* and explored a suitable time for drugs screening. The results showed that CD4^+^ T cells of CHS mice were higher compared with those in normal group. The expression of T-bet and GATA3 showed a Th1 shift and the levels of interleukin (IL)-2 and IL-4 also showed similar trend. Furthermore, IL-6 produced by T lymphocytes from CHS mice had a high level too. Then, we detected the effects of dexamethasone (DEX), cyclosporine A (CsA) and mycophenolate mofetil (MMF) on T lymphocytes *in-vitro*, and the data displayed that these immunosuppressive drugs could all inhibit the proliferation of T lymphocytes significantly. These findings suggested that T lymphocytes from CHS mice could mimic a similar immune response *in-vitro*, and it’s also a suitable method for screening anti-inflammatory and immunosuppressive agents.

## Introduction

The immune-related diseases occur in human beings while the use of clinical medicines for their treatment is accompanied by severe adverse effects, which can have serious impact on the quality of life (1). For this reason, it is important to evaluate potential of drugs to treat these diseases. The methods for evaluating the anti-inflammatory and immunosuppressive effects of new compounds mainly rely on availability of animal models, one of which is the commonly used CHS mouse model induced by DNFB (2). It is well established that CHS show a Th1 predominance combining with Th2 response, primarily with respect to cytokine secretion profiles (3, 4). Th1 cells are characterized by the production of IL-2 and IFN-*γ*, whereas Th2 cells preferentially secrete IL-4. The differentiation of Th1 and Th2 cells is regulated by the nuclear factors of T-bet and GATA3 respectively. 

It has been estimated that screening experiments of new compounds would require large number of animals. Therefore, it is very important to develop some methods for predicting anti-inflammatory and immunosuppressive effects of new compounds *in-vitro*. The proliferation of lymphocytes could be simulated by concanavalin A (ConA) or lipopolysaccharide (LPS) in orthodox *in-vitro* test (5). However, these reagents just promote the mitosis of lymphocytes, which could not reflect the real environment *in-vivo*. Therefore, we investigated the viability of T lymphocytes and the production of related cytokines to verify the immune response by a combination of* in-vivo *and* in-vitro* methods. 

## Experimental

*Mice*

Female ICR mice were purchased from PLA academy of military medical laboratory animal center (Beijing, China) at 6-8 weeks and held for 1 week to acclimatize before use. They were housed at 22–24°C with 50–60% humidity under artificial lighting conditions with a 12 h light/dark cycle. They had access to food (Huarong Animal Science and Technology Co., Ltd., Tianjin, China) and water *ad libitum*. All experiments were carried out according to the National Institutes of Health Guide for Care and Use of Laboratory Animals, and were approved by the Institutional Animal Care and Use Committee of the Medical College of the Chinese People’s Armed Police Force.

*Reagents*

DNFB, WST-1 and red blood cell lysing buffer were purchased from Sigma. IL-2, IL-4 and IL-6 enzyme linked immunosorbent assay (ELISA) kits were provided by Dakewe Biotech. Anti-mouse CD4 monoclonal antibody (mAb) was from eBioscience and fluorescein isothiocyanate (FITC) conjugated anti-rat IgG was from Boisynthesis Biotechnology. Reverse transcription polymerase chain reaction (RT-PCR) related reagents were purchased from BioTeck. DEX (Jin Yao Co., Ltd., Tianjin, NO.0812292), CsA (North China Pharmaceutical Group, NO.100701) and MMF (Roche Pharmaceutical Co., Ltd., NO.SH0409) were dissolved in saline to suitable concentration.

*Cell preparation*

The mice were sensitized with 100 μL of 0.5% (w/v) DNFB in acetone: olive oil (4:1) on shaved abdominal skin on day 0 and day 2. On day 6, mice were challenged on the dorsal and ventral surface of ears with 20 μL of 0.5% DNFB. The normal control group was painted with acetone-olive oil solutions in the same way. 24 h after elicitation, the spleens were removed aseptically and single-cell suspension was made through a 200 oculus steel rete. Purified T cells were prepared by using immunomagnetic negative selection as described previously (6). The purity of the resulting T cells population was examined by flow cytometry, and was consistently 95%. Cells were resuspended in RPMI-1640 media supplemented with 2 mmol/L L-glutamine, 10% (v/v) heat-inactivated FCS and 100 U/mL penicillin–streptomycin, and were cultured at 37 °C in a humidified atmosphere of 95 % air/5 % CO_2_.

*Proliferation assay*

For WST-1 proliferation assays, 1×10^6^ T lymphocytes were cultured in a 96 well plate for 0, 6, 12, 24, 36 and 48 h. At the end of incubation, WST-1 reagent (1:10) was added to each well and the absorption of the samples was measured 2 h after adding the reagent at 450 nm using a microplate reader.

*Immunofluorescence*

T cells were collected and washed twice. Then, cells were fixed on cover slips with 3.7% formaldehyde/PEMP for 30 min at room temperature before being washed twice with PBS and incubated with 3% (w/v) BSA in PBS (blocking solution) for one hour. Following blocking, cells were incubated for 1 day with anti-mouse CD4 mAb. The cells were washed thrice with PBS, permeabilized with 1% triton X-100 for 5 min. Cells were then incubated in secondary antibody (goat anti-rat IgG conjugated to FITC) at 37 °C for 1 h, washed thrice and covered on the coverslip with 90% glycerin in PBS. The expression of CD4 in the surface of T cells was observed with a fluorescence microscope (E800EFUV, Nikon, Japan). Then, the cells were cultured in luminescence test plates (Jet Biofil, CO., Ltd), which fluorescence intensity was detected with a microplate reader (Multiscan FC, Thermo, Finland).

*RT-PCR*

Total RNA of T cells was isolated immediately by TRIzol reagent, and the purity was assessed by the 260/280 nm ratio measurement. Equal amounts of RNA (1 μg) were reverse transcribed into cDNA using oligo (dT) primers. For PCR amplification, specific primers were synthesized by Jinsite (Nanjin, China). DNA was amplified in a 34-cycle in the following conditions: denaturation at 94°C for 30 sec and elongation at 72°C for 30 sec. The annealing temperature and primer sequences of these genes were shown in Table1. PCR products were analyzed on a 1% agarose gel. The intensity of the corresponding band was analyzed using Bio-Rad image system and Quantity One image analysis software. The amount of each gene was determined and normalized by GAPDH. 

*ELISA*

 The production of IL-2, IL-4and IL-6 secreted by T lymphocytes in supernatant was measured by ELISA according to the manufacturer’s instructions.

*Statistical analysis*

Experiments were repeated at least three times, and all data were expressed as the mean ± SD. The data were analyzed by one-way ANOVA. All statistical analyses were performed with SPSS v.13.0. p < 0.05 was considered statistically significance.

## Results

 T lymphocytes from CHS mice showed a higher viability *in-vitro*

**Figure 1:**
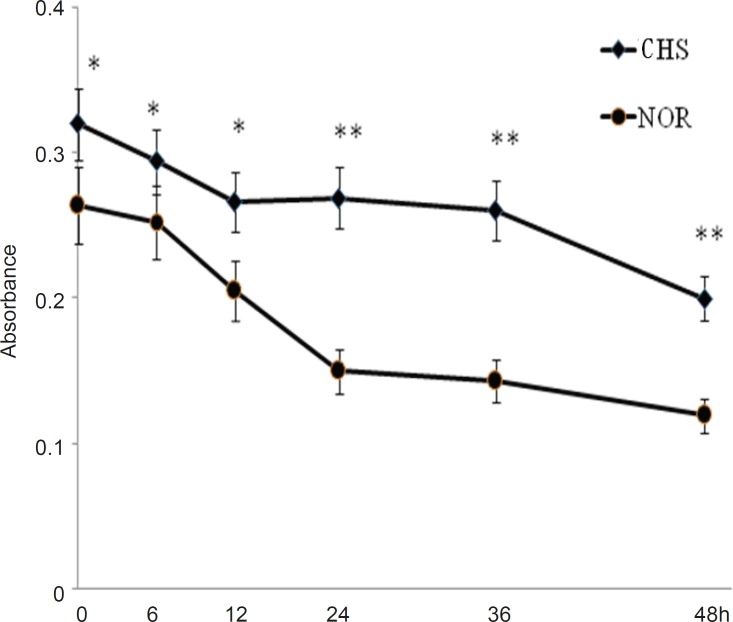
The viability of T lymphocytes *in-vitro*. The mice of CHS model were sensitized in day 0 and day 2, and then were challenged by 0.5%DNFB in day 6. The normal mice were painted with vehicle at the same way. 24 h after challenged, T cells separated from CHS and normal mice were cultured in a 96-well plate. The cell viability was detected at 0, 6, 12, 24, 36 and 48 h by WST-1 assay. Data were expressed as mean ± SD from five independent experiments. * p < 0.05, **p < 0.01 vs NOR. CHS, CHS mice; NOR, normal mice

 We first detected the viability of T cells from CHS and normal mice. As shown in Figure 1, the cells of normal mice showed a lower level of activity (0.264±0.021) compared with those of CHS mice (0.320±0.024) at the beginning (0 h). From 0 h to 48 h, T lymphocytes of CH mice maintained a relatively stable state, which suggested that T lymphocytes from CHS mice could display a higher level of activity of immune response than those from normal mice in a certain length of time.

*The expression of CD* *4 in* *T lymphocytes*

**Figure 2:**
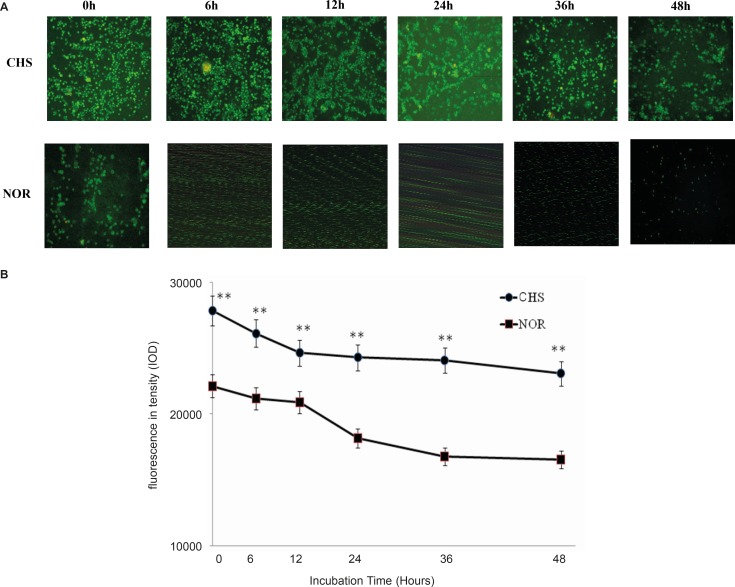
a: The expression of CD4 in T lymphocytes was observed under fluorescence microscope. CHS, CHS mice; NOR, normal mice. b: T cells were cultured in luminescence test plates, and the fluorescence intensity was detected by a microplate reader. Data were expressed as mean ± SD from triplicate tests. ** p < 0.01 vs NOR. CHS, CHS mice; NOR, normal mice

To determine whether T lymphocytes isolated from CHS mice *in-vitro* behave in the same way as those *in-vivo*, the expressions of CD4 in T lymphocytes from CHS mice and normal mice were detected by immunofluorescence. The morphological changes were observed by fluorescence microscope (Figure 2A) and fluorescence intensity was recorded by a microplate reader (Figure 2B). The results showed that the expression of CD4 in T lymphocytes from CHS mice was higher than that in normal mice.

*The expression of T-bet and GATA3*

**Figure 3:**
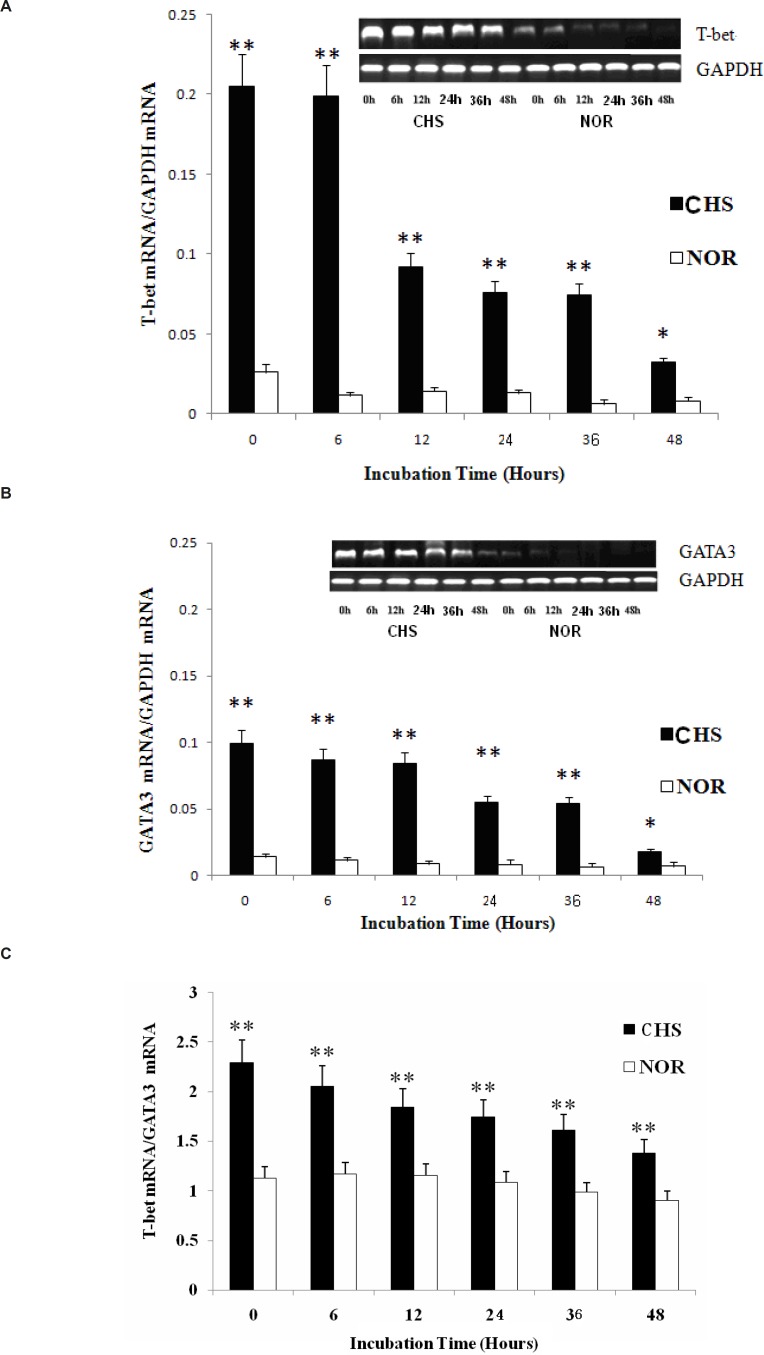
a: The expression of T-bet mRNA. Data were expressed as means ± SD from triplicate tests. * p < 0.05, **p < 0.01 vs NOR. CHS, CHS mice; NOR, normal mice. b: The expression of GATA3 mRNA. Data were expressed as means ± SD from triplicate tests. * p < 0.05, **p < 0.01 vs NOR. CHS, CHS mice; NOR, normal mice. c: The ratio of T-bet/GATA3. Data were expressed as mean ± SD from triplicate tests. * p < 0.05, **p < 0.01 vs NOR. CHS, CHS mice; NOR, normal mice

Since T-bet and GATA3 are reported to represent Th1 or Th2 immune response (4, 7), we analyzed the mRNA expression of these transcription factors to investigate whether T lymphocytes from CHS mice could maintain the Th1 dominant response *in-vitro*. The results showed that not only T-bet mRNA but also GATA3 mRNA of T cells from CHS mice showed a higher expression compared with those in normal group (Figure 3A and B). The two groups maintained a significant difference until 48 h after. Then, the T-bet/GATA3 ratio was calculated to evaluate Th polarization state (Figure 3C). The results showed a constant Th1 dominant response, indicating that T lymphocytes retained a similar trend as that *in-vivo* (8). 

*The expression and production of cytokines for Th1/Th2 cells*

**Figure 4:**
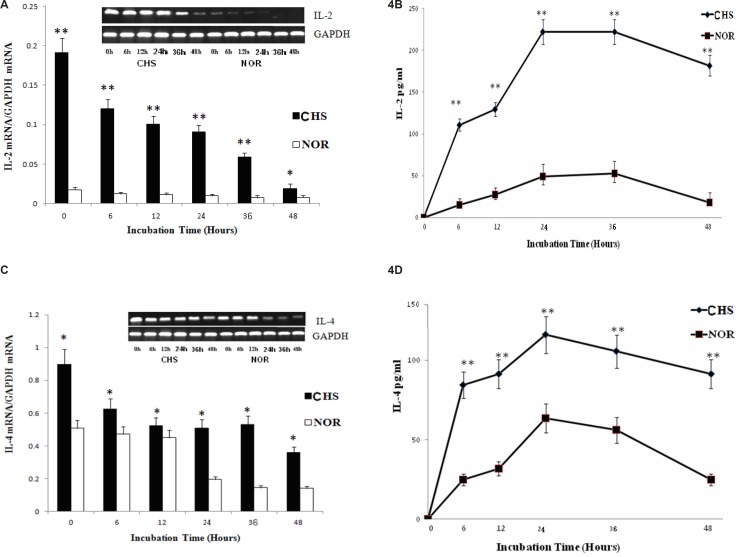
a: The expression of IL-2 mRNA. Data were expressed as mean ± SD from triplicate tests. * p < 0.05, **p < 0.01 vs NOR. CHS, CHS mice; NOR, normal mice. b: The production of IL-2. Data were expressed as mean ± SD from triplicate tests. * p < 0.05, **p < 0.01 vs NOR. CHS, CHS mice; NOR, normal mice. c: The expression of IL-4 mRNA. Data were expressed as mean ± SD from triplicate tests. * p < 0.05, **p < 0.01 vs NOR. CHS, CHS mice; NOR, normal mice. d: The production of IL-4. Data were expressed as mean ± SD from triplicate tests. * p < 0.05, **p < 0.01 vs NOR. CHS, CHS mice; NOR, normal mice

 It’s well known that the levels of IL-2 and IL-4 are the key cytokines of Th1 or Th2 cells (7). Thus, we further detected the expression and production of IL-2 and IL-4 respectively. As shown in Figure 4, the expression and production of IL-2 and IL-4 in T lymphocytes both displayed Th1 and Th2 response. The peak of IL-2 (221.8±14.8) was much higher than that of IL-4 (116.1± 12.3), which suggested a Th1 dominant response.

*The level of IL-6*

**Figure 5:**
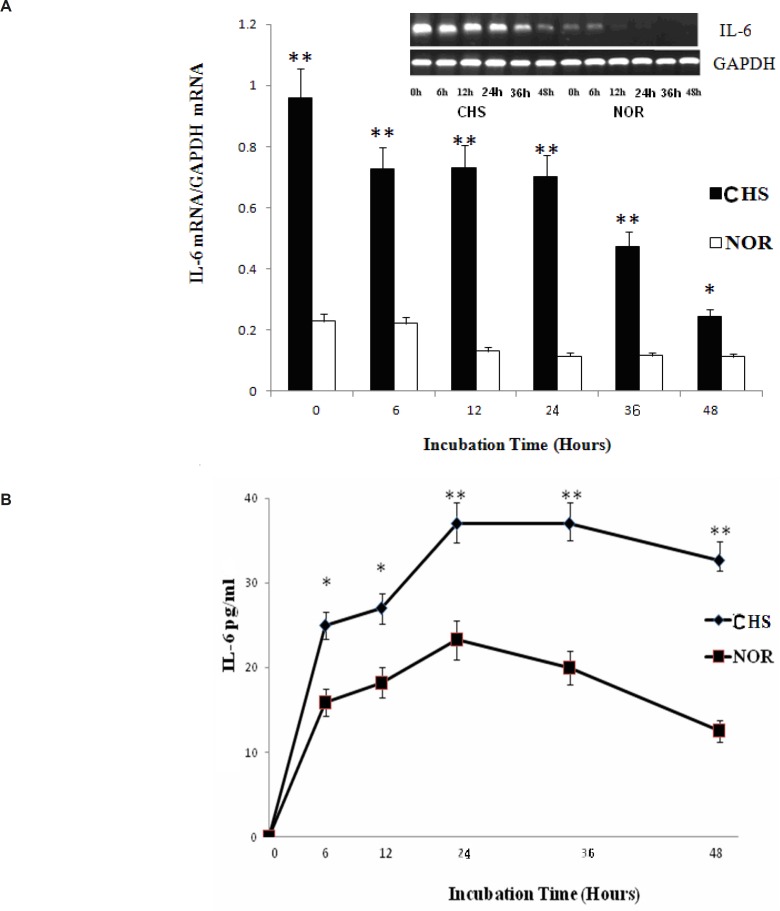
a: The expression of IL-6 mRNA. Data were expressed as mean ± SD from triplicate tests. * p < 0.05, **p < 0.01 vs NOR. CHS, CHS mice; NOR, normal mice. b: The production of IL-6. Data were expressed as mean ± SD from triplicate tests. * p < 0.05, **p < 0.01 vs NOR. CHS, CHS mice; NOR, normal mice

 IL-6 plays an irreplaceable role in inflammatory reaction of immune response (8, 9). Therefore, we chose IL-6 expression to explore the inflammatory reaction of T lymphocytes from CHS mice *in-vitro*. As shown in Figure 5, the expression of IL-6 mRNA from T lymphocytes of CHS mice was higher than those of normal group. Moreover, the production of IL-6 in CHS mice as detected by ELISA increased significantly until 48 h after. Therefore, T lymphocytes of CHS mice could exhibit inflammatory reaction *in vitro*.

*Positive drugs inhibited the proliferation of T lymphocytes in-vitro*

**Figure 6:**
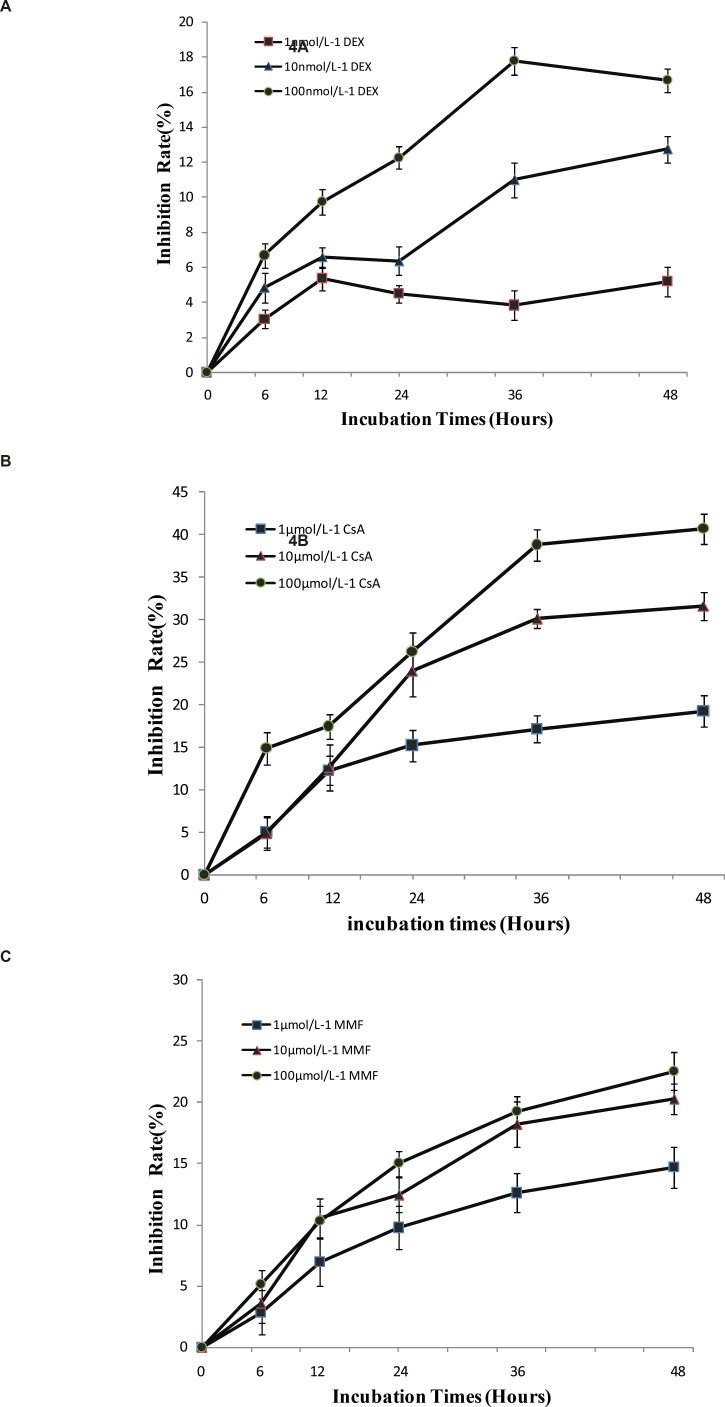
a: The effect of DEX on T lymphocytes proliferation. b: The effect of CsA on T lymphocytes proliferation. c: The effect of MMF on T lymphocytes proliferation. For all the experiments, data were expressed as mean ± SD from triplicate tests

 From the study above, it’s indicated that T lymphocytes from CHS mice could have inflammatory and immune response *in-vitro*. DEX, CsA and MMF have been used in clinics for their anti-inflammatory and immunomodulatory effects (10). Thus, T lymphocytes of CHS mice were treated with these three drugs using WST-1 assay. The data indicated that these drugs could inhibit T lymphocytes proliferation in a dose and time-dependent manner *in-vitro* (Figure 6), which suggested that T lymphocytes of CHS mice were suitable for the detection of immunosuppressive drugs.

**Table 1:** Primer sequences and annealing conditions in this study

**Gene name**	**Primer sequence**	**Annealing temperature**
GAPDH	Forward:5’-TGGAGAAACCTGCCAAGTATG-3’ Reverse: 5’-CCCTGTTGCTGTAGCCGTAT-3’	56°C
GATAT3	Forward:5’- AGTGTGTGAACTGCGGGGCA -3’ Reverse: 5’- TCCAGCGCGTCATGCACCTT -3’	57°C
IFN-γ	Forward:5’- GCTTCTCCTCCTGCGGCCTA -3’ Reverse: 5’- GGCTCTGCAGGATTTTCATGTCA -3’	55°C
IL-2	Forward:5’- GACACTTGTGCTCCTTGTCA -3’ Reverse: 5’- TCAATTCTGTGGCCTTGCTTG -3’	58°C
IL-4	Forward:5’- TCGGCATTTTGAACGAGGTC -3’ Reverse: 5’- GAAAAGCCCGAAAGAGTCTC -3’	58°C
IL-6	Forward:5’-CCTCTCTGCAAGAGACTTCCATC-3’ Reverse: 5’-AGCCACTCCTTCTGTGACTCCAG-3’	59°C
T-bet	Forward:5’- GATCGTCCTGCAGTCTCTCC -3’ Reverse: 5’- AACTGTGTTCCCGAGGTGTC -3’	57°C

## Discussion

It is well known that the results of animal experiments can be a true reflection of the drug therapeutic effect *in-vivo*. However, the differences between individual animals need to increase the number of samples, which means that the screening experiments of large-scale drugs would require large number of animals. Therefore, the use of *in-vitro* tests to evaluate drugs is an effective and easy way. Traditional methods use agonists like phytohaemagglutinin (PHA), ConA, LPS or pokeweed mitogen (PWM) to activate lymphocytes (11). Nevertheless, these reagents couldn’t really reflect immune response *in-vivo*. 

CHS is a T-cells–mediated cutaneous inflammatory reaction caused by the repeated skin exposure to contact allergens. The important qualitative aspects of immune responses to CHS relate to the differential development of functional subpopulations of T lymphocytes. Mosmann and Coffman (12) defined two distinct CD4^+^T cells (Th1 and Th2 cells) in terms of their differential cytokine-producing patterns. As shown in murine models, topical exposure of mice to DNFB will induce a selective Th1 type reaction. Previous studies have found that the reaction showed a decline phenomenon after 72 h *in-vivo* (13). If T lymphocytes of CHS mice could be able to maintain the immune response *in-vitro* for a certain time, they would be used for the evaluation of new compounds. Therefore, first we detected the proliferation of T lymphocytes and the expression of CD4 from 0 to 48 h. The results revealed a higher viability of T lymphocytes from CHS mice *in- vitro*. In addition, the expression of CD4^+^ T lymphocytes in CHS mice was higher than that in normal mice *in-vitro*. 

The balance between the Th1- and Th2-dominant immunity (Th1/Th2 balance) is thought to be important for the development and maintenance of various immune disorders (14). By analyzing the levels of IL-2 and IL-4 between CHS and normal group, we confirmed that T lymphocytes in CHS mice could show a significant Th1 type immune response *in-vitro*. Furthermore, the differentiation of Th cells towards Th1 or Th2 cells is regulated by T-bet or GATA3 (15), whose ratio could represent Th1/Th2 balance (16). In the present study, the ratio of T-bet/GATA3 showed that the Th1 cells played a dominant role *in-vitro*, which were consistent with previous studies *in-vivo* (3). 

Increasing evidence has indicated that the mouse model of CHS acts specifically and leads to the formation of inflammatory immune disorders. IL-6 is a major mediator to enhance the production of acute phase proteins in skin as a second signal required for T-cell proliferation. The expression and production of IL-6 from T lymphocytes of CHS mice was significantly higher than that in normal group. Therefore, we can speculate that T lymphocytes of CHS mice could represent a stable inflammatory reaction *in-vitro*.

According to these data shown above, T lymphocytes of CHS mice could be a suitable model for evaluation of anti-inflammatory and immunosuppressive effects of drug *in vitro*. The anti-inflammatory actions of glucocorticoids, including DEX, are primarily attributed to the suppression of factors essential for transcription of cytokines and chemokine mRNAs (17). CsA could bind to the cytosolic protein cyclophilin, particularly in T-lymphocytes, and then inhibit the synthesis of a number of cytokines, particularly IL-2 (18). MMF is a selective inhibitor of inosine 5›-monophosphate dehydrogenase, leading to inhibition of the function of T cells (19, 20). Therefore, DEX, CsA and MMF were selected to explore their effects on T lymphocytes proliferation. Our data indicated that these drugs could strongly suppress the proliferation of T lymphocytes from CHS mice, which suggested that other immunosuppression compounds might have similar effects on T lymphocytes *in-vitro*. 

In conclusion, T lymphocytes isolated from CHS mice may be a novel *in-vitro* model in screening anti-inflammatory and immunosuppressive drugs. Meanwhile, it’s also a suitable method for investigating the target or pathway of new compounds on Th1 dominant immune disease.

## References

[1] Drigo I, Piscianz E, Valencic E, De Iudicibus S, Tommasini A, Ventura A, Decorti G (2010). Selective resistance to different glucocorticoids in severe autoimmune disorders. Clin. Immunol.

[2] Kammeyer A, Bos JD, Teunissen MB (2008). Teunissen. Postelicitation model of allergic contact dermatitis for predicting the efficacy of topical drugs. Exp. Dermatol.

[3] Hayashi T, Hara S, Hasegawa K (2009). Enhanced contact hypersensitivity by delayed T-helper 2 response in BALB/c mice. Allergy Asthma Proc.

[4] Ishizaki K, Yamada A, Yoh K, Nakano T, Shimohata H, Maeda A, Fujioka Y, Morito N, Kawachi Y, Shibuya K, Otsuka F, Shibuya A, Takahashi S (2006). Th1 and Type 1 Cytotoxic T Cells dominate responses in T-bet overexpression transgenic mice that develop contact dermatitis. J. Immunol.

[5] Zhou YB, Ye RG, Li YJ, Xie CM, Wu YH (2008). Effect of anti-CD134L mAb and CTLA4Ig on ConA-induced proliferation, Th cytokine secretion, and anti-dsDNA antibody production in spleen cells from lupus-prone BXSB mice. Autoimmunity.

[6] Li T, Fan GX, Wang W, Li T, Yuan YK (2007). Resveratrol induces apoptosis, influences IL-6 and exerts immunomodulatory effect on mouse lymphocytic leukemia both in-vitro and in-vivo. Int Immunopharmacol.

[7] Rengarajan J, Szabo SJ, Glimcher LH (2000). Transcriptional regulation of Th1/Th2 polarization. Immunol. Today.

[8] Bonneville M, Chavagnac C, Vocanson M, Rozieres A, Benetiere J, Pernet I, Denis A, Nicolas JF, Hennino A (2007). Skin contact irritation conditions the development and severity of allergic contact dermatitis. J. Invest. Dermatol.

[9] Sakai S, Sugawara T, Kishi T, Yanagimoto K, Hirata T (2010). Effect of glucosamine and related compounds on the degranulation of mast cells and ear swelling induced by dinitrofluorobenzene in mice. Life Sci.

[10] Collinge M, Cole SH, Schneider PA, Donovan CB, Kamperschroer C, Kawabata TT (2010). Human lymphocyte activation assay: An in-vitro method for predictive immunotoxicity testing. J. Immunotoxicol.

[11] Eisenthal A, Marder O, Dotan D, Baron S, Lifschitz-Mercer B, Chaitchik S, Tirosh R, Weinreb A, Deutsch M (1996). Decrease of intracellular fluorescein fluorescence polarization (IFFP) in human peripheral blood lymphocytes undergoing stimulation with phytohaemagglutinin (PHA), concanavalin A (ConA), pokeweed mitogen (PWM) and anti-CD3 antibody. Biol. Cell.

[12] Mosmann TR, Coffman RL (1989). TH1 and TH2 cells: different patterns of lymphokine secretion lead to different functional properties. Annu. Rev. Immunol.

[13] Desvignes C, Etchart N, Kehren J, Akiba I, Nicolas JF, Kaiserlian D (2000). Oral Administration of Hapten Inhibits In Vivo Induction of Specific Cytotoxic CD8+ T Cells Mediating Tissue Inflammation A Role for Regulatory CD4+ T Cells. J. Immunol.

[14] Zheng J, Liu Y, Qin G, Lam KT, Guan J, Xiang Z, Lewis DB, Lau YL, Tu W (2011). Generation of human Th1-like regulatory CD4+ T cells by an intrinsic IFN-γ- and T-bet-dependent pathway. Eur. J. Immunol.

[15] Chakir H, Wang H, Lefebvre DE, Webb J, Scott FW (2003). T-bet/GATA-3 ratio as a measure of the Th1/Th2 cytokine profile in mixed cell populations: predominant role of GATA-3. J. Immunol. Methods.

[16] Szabo SJ, Sullivan BM, Stemmann C, Satoskar AR, Sleckman BP, Glimcher LH (2002). Distinct effects of T-bet in TH1 lineage commitment and IFN-g production in CD4 and CD8 T cells. Science.

[17] De Bosscher K, Haegeman G (2009). Minireview: Latest perspectives on anti-inflammatory actions of glucocorticoids. Mol. Endocrinol.

[18] Lee CL, Jiang PP, Sit WH, Wan JM (2007). Proteome of human T-lymphocytes with treatment of cyclosporine and polysaccharopeptide: Analysis of significant proteins that manipulate T-cells proliferation and immunosuppression. Int. Immunopharmacol.

[19] Mehling A, Grabbe S, Voskort M, Schwarz T, Luger TA, Beissert S (2000). Mycophenolate mofetil impairs the maturation and function of murine dendritic cells. J. Immunol.

[20] Orvis AK, Wesson SK, Breza TS Jr, Church AA, Mitchell CL, Watkins SW (2009). Mycophenolate mofetil in dermatology. J. Am. Acad. Dermatol.

